# Characterization of the complete mitochondrial genome of *Illeis bistigmosa* (Coleoptera: Coccinellidae)

**DOI:** 10.1080/23802359.2022.2057244

**Published:** 2022-04-05

**Authors:** Guoyuan Zhu, Yongke Zhang, Bo Duan, Zhonghua Wu, Jinqiang Wang, Hongchang A, Zubing Zhang, Yu Zhang

**Affiliations:** a Yunnan Institute of Tropical Crops, Jinghong, China; b School of Plant Protection, Hainan University, Haikou, China

**Keywords:** *Illeis bistigmosa*, mitochondrial genome, phylogenetic tree

## Abstract

*Illeis bistigmosa* (Mulsant, 1850) is a potential biological control agent of powdery mildews. We have determined the first mitochondrial genome of *I. bistigmosa*. The circular mitogenome of *I. bistigmosa* consists of 17,840 bp including 13 protein-coding genes, 22 tRNAs, 2 rRNAs, and a control region (D-loop). The base composition was AT-biased (78.44%). Maximum-likelihood phylogenetic trees strongly supported the monophyly of Coccinellinae. *Illeis bistigmosa* is the sister group of *Halyzia sedecimguttata* and Halyziini species (unclassified Halyziini), within fungivorous coccinellids. *Illeis bistigmosa* mitochondrial genome will be a fundamental resource for understanding the molecular phylogenetic relationships of the species-rich family Coccinellidae of Coleoptera.

*Illeis bistigmosa* (Mulsant, 1850) belongs to the Coccinellid tribe Halyziini (=Psylloborini) (Pakaluk et al. 1994) and is a medium-sized lady beetle (length: 3.63–5.33 mm; width: 2.46–4.16 mm; elytra very weakly convex; winged, yellowish, glabrous), distributed in China, India, Indonesia, Malaysia, Nepal, Philippines, Sri Lanka, Thailand, and Vietnam. It mainly feeds on spores and mycelia of powdery mildews. It plays a vital ecological function in minimizing the impact of powdery mildew infections due to its wide distribution in nature, robust reproduction ability, long lifetime, huge feeding volume, and ability to consume a variety of powdery mildew (Timberlake 1943; Ghorpade 1976; Krishnakumar and Maheswari 2004). Previous research on *I. bistigmosa* mostly focused on its morphology and biology (Ghorpade 1976; Krishnakumar and Maheswari 2004; Zhu et al. 2020), but the species’ evolutionary relationships were unclear. The complete mitochondrial genome of *I. bistigmosa* was analyzed to provide new insight into the phylogeny of the Coccinellidae.

In this study, ladybird samples were collected from a Rubber tree infested with powdery mildew at Jinghong, Yunnan Province, China (22.0260 N, 100.7860 E) in 2020, and were subsequently identified to species by morphology. The voucher specimens were deposited at the Research Center of Plant Protection and Microbial Utilization, Yunnan Institute of Tropical Crops (http://www.yitc.com.cn/, Guoyuan Zhu, zhuguoyuan89@126.com) under the voucher number Sjpc-xj-1. DNA was extracted from 6 *I. bistigmosa* insects collected from the same place and same plant. The DNA sequencing was performed using Illumina NovaSeq 6000 in BIOZERON Biotechnology Co., Ltd. (Shanghai, China). Raw and clean reads were assembled by SPAdes v3.14.1 (Bankevich et al. 2012). The assembled contigs were compared with the mitochondrial genome of related species using blastn version (BLAST 2.2.30+). The MITOS web server and CGView server were used for gene annotation and mapping graphic view of the mitogenomes, respectively (Grant and Stothard 2008; Bernt et al. 2013).

The complete mitochondrial genome of *I. bistigmosa* was submitted to Genbank (accession number MZ325765). It is a closed circular molecule of 17,840 bp in length containing 13 protein-coding genes (PCGs), 22 transfer RNA (tRNA) genes, 2 ribosomal RNA (rRNA) genes, and 1 A + T-rich region. The tRNA genes length ranged between 55 and 70 bp, the 2 rRNA genes were 1357 bp and 744 bp in length, respectively, and the 13 PCGs had 11,053 bp overall length. The H-strand has an overall base composition of 41.45% A, 8.94% G, 12.61% C, and 36.99% T, with a remarkable 78.44% A + T bias. 4 tRNA genes (*trnF*, *trnG*, *trnR*, and *trnW*) lacked TψC loop, *trnS1* lacked dihydrouracil arm and loop, and *trnP* lacked the TψC arm. The mitochondrial genes were encoded on the heavy strand except for 4 PCGs, 2 rRNA genes, and 8 tRNA genes. All protein-encoding gene initiation codons were ATN apart from *cox1* (TTG). 9 of 13 protein-coding genes terminate with complete stop codons, TAA (*atp8*, *cob*, *cox1*, *nad2*, *nad3*, *nad4l*, and *nad6*) and TAG (*atp6* and *nad1*), whereas 4 genes ended with incomplete stop codons, T−.

The phylogenetic analysis was carried out using 13 protein-coding genes of *I. bistigmosa* and the other 12 coccinellids. Meanwhile, *Henosepilachna vigintioctopunctata* and *Henosepilachna pusillanima* were chosen as outgroups. The phylogenetic tree was built with the maximum-likelihood analysis with 1000 bootstrap replicates through IQ-TREE (Nguyen et al. 2015). In the tree, the members of the same species or genus were grouped with high bootstrap confidence values. In terms of topology, the species referred here could be divided into two clades from the consensus trees: Epilachnini coccinellids clustered together to form the first clade branching from the base of the tree; Coccinellini coccinellids and Halyziini coccinellids clustered together to form the second clade. It showed that phylogeny may be related to the diet of coccinellids. Species with similar food preferences all clustered together in the second clade. *Illeis bistigmosa* and the other two Halyziini species clustered into a branch, and formed a sister group with predatory coccinellids, indicating that they were closely related in this phylogeny (Figure 1). However, the monophyly of predatory coccinellids was not supported by sufficient bootstrap values. The results provide evidence that phylogeny may be related to the diet of coccinellids, but more details are required to identify.

**Figure 1.**
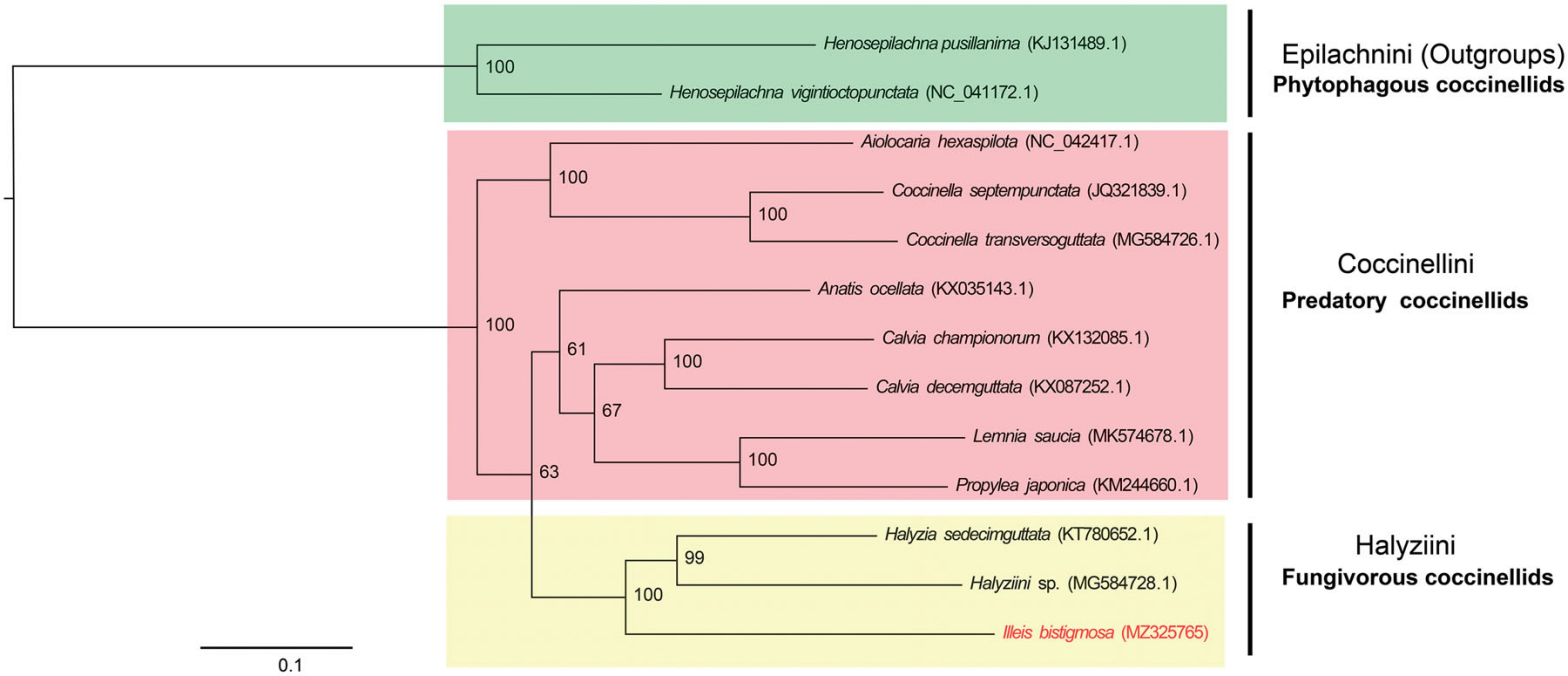
Phylogenetic tree of *I. bistigmosa* and 12 other species, with *H. pusillanima* and *H. vigintioctopunctata* as out-groups. ML bootstrap values (1000 replications) are indicated in front of each node.

## Authors’ contributions

Guoyuan Zhu: Conceptualization, Investigation, Data Curation, Formal analysis, Writing - Original Draft; Yongke Zhang (Corresponding Author): Funding Acquisition, Supervision, Visualization, Writing - Review & Editing; Bo Duan: Investigation, Formal analysis, Writing - Review & Editing; Zhonghua Wu: Investigation, Data Curation; Jinqiang Wang: Resources, Supervision, Project administration; Hongchang A: Investigation; Zubing Zhang: Investigation. Yu Zhang: Resources. All authors agree to be accountable for all aspects of the work.

## Data Availability

The data that support the findings of this study are openly available in GenBank of NCBI at (https://www.ncbi.nlm.nih.gov/) under the accession no. MZ325765. The associated BioProject, SRA, and Bio-Sample numbers are PRJNA732568, SRR14949257, and SAMN19328177, respectively.
